# Nontuberculous mycobacteria in China: incidence and antimicrobial resistance spectrum from a nationwide survey

**DOI:** 10.1186/s40249-021-00844-1

**Published:** 2021-04-29

**Authors:** Chun-Fa Liu, Yi-Meng Song, Wen-Cong He, Dong-Xin Liu, Ping He, Jing-Jing Bao, Xin-Yang Wang, Yan-Ming Li, Yan-Lin Zhao

**Affiliations:** 1 National Center for Tuberculosis Control and Prevention, Chinese Center for Disease Control and Prevention, Changbai Road 155, Changping, Beijing102206 China; 2 National Center of Gerontology, Beijing Hospital, Dongdandahua Road 1, Dongcheng, Beijing, 100730 China; 3 National Institute for Communicable Disease Control and Prevention, Chinese Center for Disease Control and Prevention, Beijing, 102206 China; 4 Inner Mongolia Medical University, Inner Mongolia, 010110 China; 5 Department of Basic Medicine, Harbin Medical University, Heilongjiang, 150081 China; 6 National Clinical Research Center for Infectious Disease, Shenzhen Third People’s Hospital, Guangdong, 518112 China

**Keywords:** Nontuberculous mycobacteria, Pulmonary disease, Prevalence, Drug resistance

## Abstract

**Background:**

Information on the prevalence and resistance spectrum of nontuberculous mycobacteria (NTM) in China is mainly based on regional or local data. To estimate the proportion of NTM cases in China, a national survey of NTM pulmonary disease was carried out based on acid-fast positive sputum samples collected in 2013.

**Methods:**

Sputum samples collected from enrolled presumptive cases in 72 nationwide tuberculosis surveillance sites from the 31 provinces in the mainland of China were cultured using L-J medium at the National tuberculosis reference laboratory (NTRL). MALDI-TOF MS identified the species of re-cultured strains, and minimal inhibitory concentrations (MICs) were determined to evaluate the drug susceptibility of NTM isolates. Data analysis used statistical software SPSS version 22.0 for Windows statistical package.

**Results:**

Of 4917 mycobacterial isolates cultured, 6.4% [317/4917, 95% confidence interval (*CI*) 5.8%–7.2%] were confirmed as NTM, among which 7.7% (287/3709, 95% *CI* 6.9%–8.6%) were from the southern region. In inland and coastal China, 87.7% (95% *CI* 78.7%–93.2%) and 50.0% (95% *CI* 43.7%–56.3%) of isolates, respectively, were slow-growing mycobacteria (SGM), with the remaining rapid growing mycobacteria (RGM). A total of 29 species were detected, *Mycobacterium abscessus* had higher clarithromycin-inducible resistance rates than *M. massiliense* (65.67% vs 2.22%). *M. kansasii* presented lower resistance rates in linezolid and moxifloxacin than *M. avium-intracellulare* complex (3.23% vs 66.67%, 0 vs 47.22%) and other SGM (3.23% vs 38%, 0 vs 26%).

**Conclusions:**

More NTM pulmonary disease was observed in the south and coastal China (*P* < 0.01). SGM was widely distributed, and more RGM are present in southern and coastal China (*P* < 0.01). The antimicrobial resistance spectrum of different NTM species was significantly different and accurate species identification would be facilitated to NTM pulmonary disease treatment.
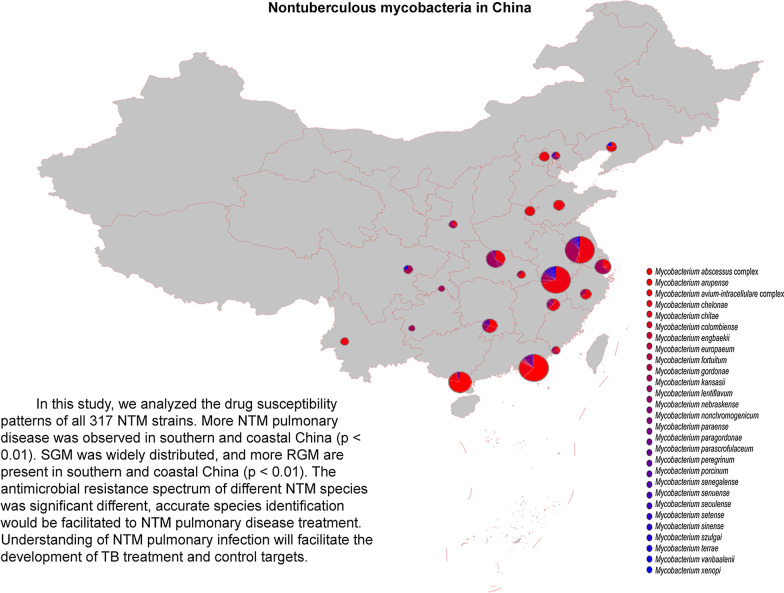

## Background

Nontuberculous mycobacteria (NTM) pulmonary disease (NTM-PD) is a severe progressive illness caused by non-tuberculous mycobacterium, requiring complicated treatment with multiple anti-mycobacterial drugs for more than 12 months [1]. Several studies have reported increased incidences of pulmonary NTM isolation, likely due to socioeconomic and medical progress, including advances in radiological diagnostics, which have improved the rate of detection of pulmonary abnormalities [2]. However, the increase in the aging population with chronic lung diseases or who are immunocompromised may have also contributed to the emergence of NTM-PD. Mortality of NTM-PD is higher than that attributable to *Mycobacterium tuberculosis* (MTB) due to inappropriate treatment and high rates of therapy failure [3]. A meta-analysis indicted the proportion of NTM among mycobacterial isolates in China was higher than most regions in the whole world except that in Northern India [4]. Although several NTM epidemiology studies in China have been reported, the NTM isolates were primarily obtained from medical institutions and comprised incomplete samples [3, 5]. Therefore, precise incidence and prevalence data are not available.

Antimicrobial agent drug susceptibility testing (DST) is essential for the treatment of infections, and is essential for guiding clinical treatment. Besides, some acid-fast staining bacteria those are not mycobacteria, such as *Gordonia* and *Nocardia*, have similar colony morphology on Lowenstein-Jensen (L-J) media. Furthermore, most NTM is inherently resistant to standard anti-tuberculosis drugs, and different species exhibit varying resistance phenotypes [6]. Therefore, an accurate and rapid method for NTM identification is essential. Because of the differences in local development, the national information associated with NTM prevalence and resistance spectrum in China was obtained by meta-analysis that data was collected in regional areas, which leads to a lack of suitable species identification and DST [4, 7].

In this study, matrix-assisted laser desorption ionization–time of flight mass spectrometry (MALDI-TOF MS), a technology with low time consumption, modest costs, and high accuracy [8, 9], was used to screening NTM from cultured acid-fast clinical strains isolated from sputum. MICs detected by the broth microdilution method were measured for RGM and SGM among the NTM isolates. The objectives of this study were to determine the proportion of NTM in isolated mycobacteria and determine their susceptibility to various antimicrobial drugs recommended by the United States Clinical and Laboratory Standards Institute (CLSI) in the mainland of China.

## Methods

### Study subjects

**Fig. 1:**
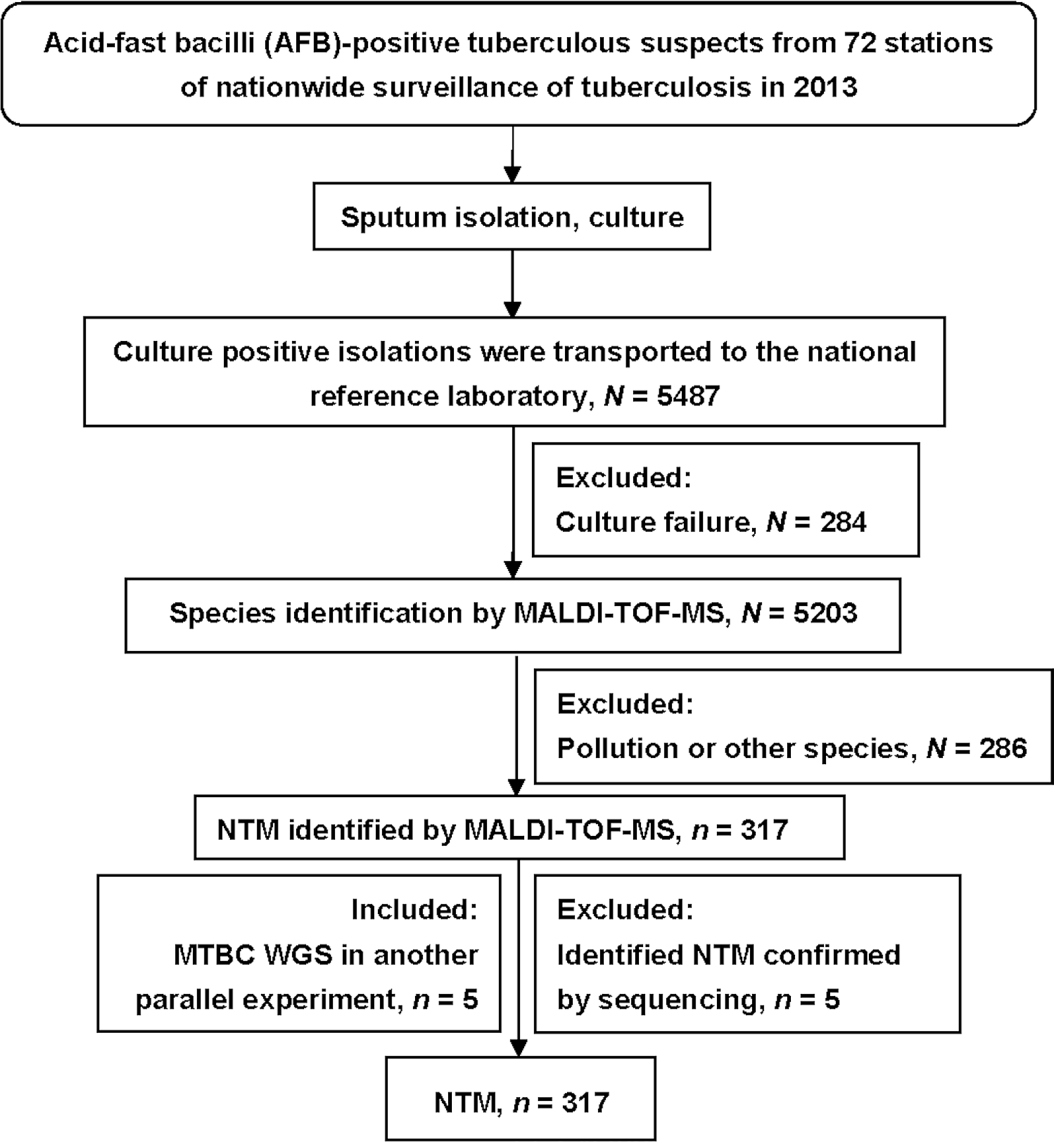
Diagram of NTM identification. *NTM* nontuberculous mycobacteria, *MALDI-TOF MS* matrix-assisted laser desorption ionization–time of flight mass spectrometry, *MTBC* mycobacterium tuberculosis complex, *WGS* whole genome sequencing, *CI* confidence interval

A total of 5487 isolates from patients with suspected acid-fast bacilli-positive tuberculous were collected at 72 nationwide tuberculosis surveillance stations in 2013 and sent to NTRL (Fig. 1). At least one site among the 72 centers from which strains collected was in each of the 31 provinces and municipalities of China. The number of centers assigned to each province and municipality was proportional to the number of new smear-positive cases reported by that province, relative to the previously reported total number of cases nationwide [10].

### Study process

All strains were re-cultivated using L-J medium in NTRL; species identification was used to culture-positive strains by MALDI-TOF MS after four weeks. Identified NTM species were confirmed by sequencing partial genes (including *16S* rRNA, *hsp*65, *rpoB*, and *ITS*). DSTs were performed using the broth microdilution method, with Sensititre® SLOMYCO plates for SGM and a panel of 13 drugs, and Sensititre® RIPMYCOI plates and 15 drugs for RGM (TREK Diagnostic Systems, Cleveland, USA). Using an Ultrasonic Milling Instrument (TB Healthcare, China), 0.5 McFarland bacterial suspensions were prepared from colonies grown on L-J culture medium. Suspensions were diluted 100-fold by adding 100 μl of 0.5 McFarland suspensions to 10 ml of Mueller–Hinton broth, with or without oleic acid-albumin-dextrose-catalase. Aliquots (100 μl) of standard 1.5 × 10^5^ CFU/ml inoculum were distributed into each well using a semi-automated Sensititre® Auto-inoculator (Thermo Fisher, Scientific Inc., USA). To prevent evaporation and skipped wells during incubation, plates were accurately and adequately sealed with adhesive membranes and incubated at the recommended temperature [11]. MIC was defined as the lowest concentration without apparent visible bacteria growth compared with positive controls and was measured by two readers, aided by a Vizion™ Digital viewing system. *Mycobacterium abscessus* (ATCC 19977) and *Mycobacterium intracellulare* (ATCC 13950) were used as quality control samples in every batch of DSTs. RGM were classified as susceptible, intermediate, or resistant, depending on the MIC values obtained, according to the CLSI criteria [12]. The current standard (CLSI M24, 3rd edition) [11] includes recommendations for DST of SGM, including the MAC, *M. kansasii*, and SGM other than MAC and *M. kansasii*.

### Statistical analysis

The statistical software IBM SPSS Statistics version 22.0 (SPSS, Inc., Chicago, IL, USA) was used for data analysis. According to the Wilson procedure, the 95% confidence interval for proportions was calculated with correction for continuity, as described by Robert Newcombe, derived from a procedure outlined by E. B. Wilson [13]. Pearson Chi-square Test tested all theoretical numbers *T*  ≥  5 and total sample size *N* ≥ 40. If the theoretical number *T*  <  5 but *T*  ≥  1, and *N*  ≥  40, the Continuous Corrected Chi-square Test of Association is used for testing. If there are theoretical numbers *T*  <  1 or *N*  <  40, Fisher Exact Probability Test will be used. *P*  <  0.01 defined as statistically significant.

## Results

### The ratio of pulmonary NTM diseases

A total of 5487 isolates were cultivated using L-J medium; recovery failed for 284 isolates. MALDI-TOF MS identified all cultured isolates and 286 strains were not mycobacteria. Finally, we obtained 317 NTM isolates from 4917 mycobacterium strains, according to MALDI biotyping results. NTM species were confirmed by partial gene sequencing (including 16S rRNA, *hsp65*, *rpoB*, and *ITS*). Five strains were identified as *Mycobacterium tuberculosis* complex (MTBC). Another five strains identified as MTBC by MALDI-TOF MS were classified as NTM based on whole gene sequencing in another ongoing research program. Finally, we identified 317 NTM strains from 31 provinces in China (Fig. 1).

**Fig. 2:**
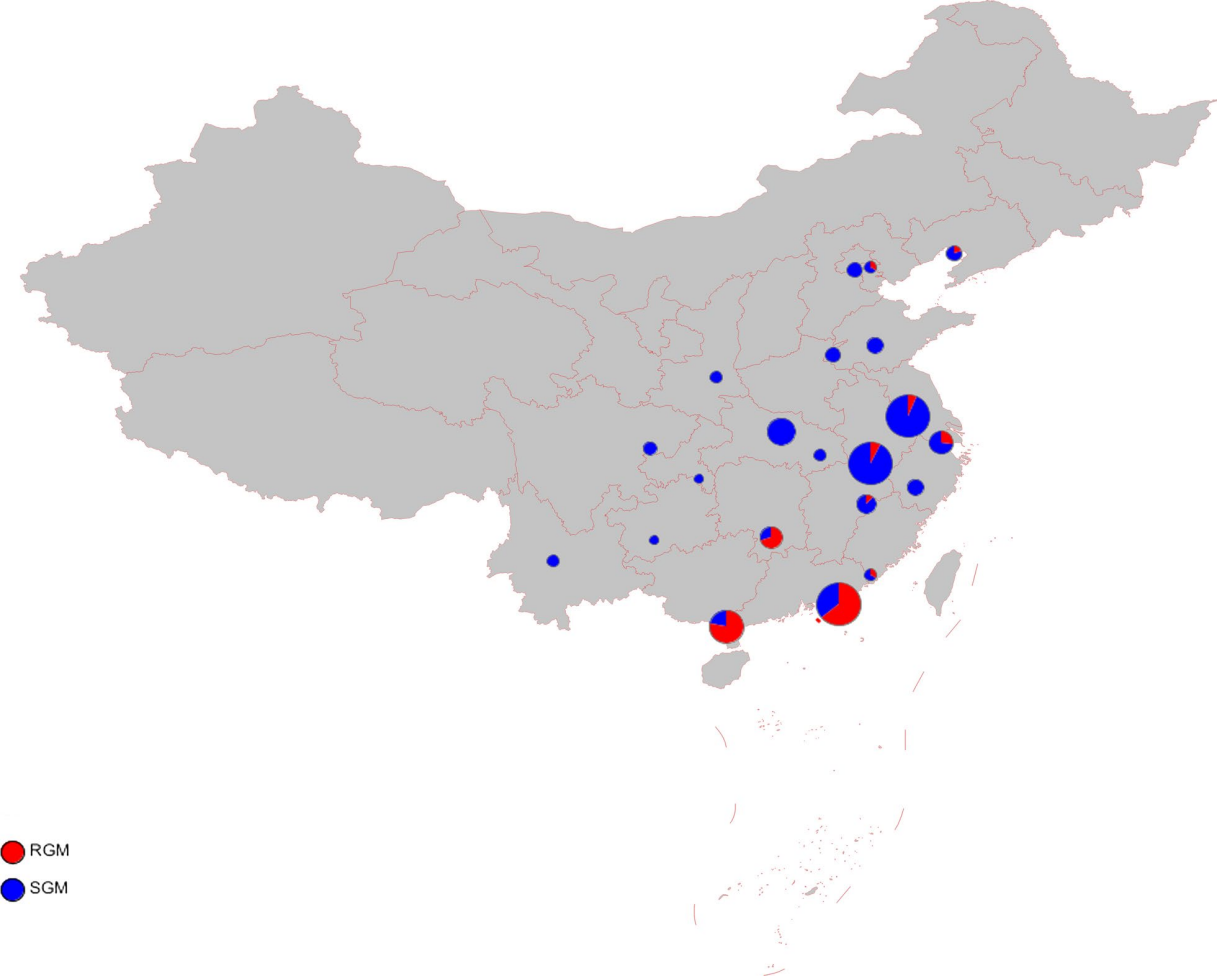
Distribution of RGM and SGM in different province of China. *RGM* rapid growing mycobacteria, SGM slow growing mycobacteria

As shown in Table 1, of 317 NTM strains, 287 (7.7%, 95% *CI* 6.9%–8.7%) of total isolates were detected in the southern region, and the NTM infection rate in the southern region was higher than that in the northern region (7.7% vs 2.5%, *P* < 0.01). In stratified analysis, the ratio of NTM infection varied according to a geographic area (Table 1): the higher ratio was 10.7% (95% *CI* 9.5%–12.1%) for the coastal region than 3.0% (95% *CI* 2.4%–3.7%) in the inland area. Prevalence rates in individual provinces are presented in Fig. 2. The composition ratio of RGM to SGM in northern and southern China differed markedly (*P*  <  0.01). Significantly more RGM were present in coastal than inland China (*P*  <  0.01).

**Table 1:** Prevalence of NTM infection in different regions based on geographic areas of China

Regions	Isolates	NTM		NTM Rate (95% *CI*)		*P*-value
		RGM	SGM	RGM Rate (95% *CI*)	SGM Rate (95% *CI*)	
South	3709	287		7.7 (6.9‒8.6)		< 0.01
		126	161	43.9 (38.3‒49.7)	56.1 (50.3‒61.7)	< 0.01
North	1208	30		2.5 (1.7‒3.5)		
		2	28	6.7 (1.9‒2.1)	93.3 (78.7‒98.2)	
Coastal	2207	236		10.7 (9.5‒12.1)		< 0.01
		118	118	50.0 (43.7‒56.3)	50.0 (43.7‒56.3)	< 0.01
Inland	2710	81		3.0 (2.4‒3.7)		
		10	71	12.4 (6.9‒21.3)	87.7 (78.7‒93.2)	

Provinces locate in northern region: Hebei, Beijing, Tianjin, Liaoning, Shanxi, Shandong, Henan, Shaanxi, Inner Mongolia, Jilin, Heilongjiang, Tibet, Gansu, Qinghai, Ningxia, Xinjiang

Provinces locate in southern region: Yunnan, Sichuan, Guizhou, Chongqing, Shanghai, Jiangsu, Zhejiang, Fujian, Anhui, Jiangxi, Hunan, Hubei, Guangxi, Guangdong, Hainan

Provinces locate in coastal region: Tianjin, Liaoning, Shanghai, Jiangsu, Zhejiang, Fujian, Shandong, Guangdong, Guangxi, Hainan, Hebei

Provinces locate in inland region: Beijing, Shanxi, Inner Mongolia, Jilin, Heilongjiang, Anhui, Jiangxi, Henan, Hubei, Hunan, Chongqing, Sichuan, Guizhou, Yunnan, Tibet, Shaanxi, Gansu, Qinghai, Ningxia, Xinjiang

*NTM* nontuberculous mycobacteria, *RGM* rapid growing mycobacteria, *SGM* slow growing mycobacteria

### The spectrum of NTM species

**Fig. 3:**
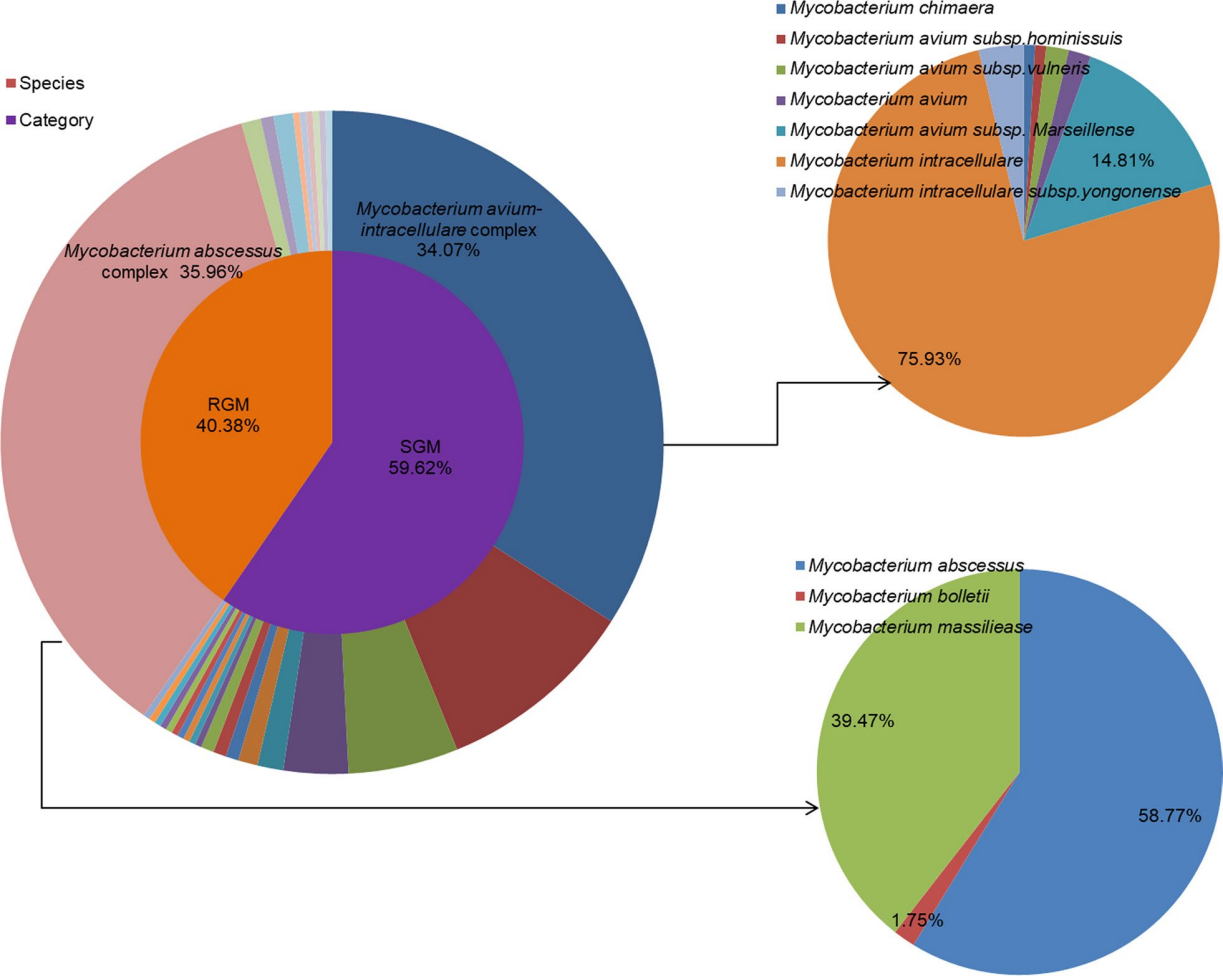
Species distribution among the NTM isolates form the mainland of China. RGM: Rapid growing mycobacteria, SGM: Slow growing mycobacteria

As shown in Fig. 3, 29 species were detected, including 19 SGM and 10 RGM; 27 species were detected in southern China and observed only seven species in northern China. The five most frequently isolated NTM, accounting for 88.5% of all NTM species, belonged to the *Mycobacterium abscessus* complex (MABC) (36.0%, 95% *CI* 30.7%‒41.5%), the *Mycobacterium avium-intracellulare* complex (MAC) (34.1%, 95% *CI* 28.9%‒39.6%), *M. kansasii* (9.8%, 95% *CI* 6.8%‒13.7%), *M. paragordonae* (5.4%, 95% *CI* 3.3%‒8.6%), and *M. lentiflavum* (3.2%, 95% *CI* 1.6%‒5.9%). The MAC included seven subspecies: *M. avium* subsp. *hominissuis* (1 isolate), *M. avium* subsp. *vulneris* (2 isolates), *M. avium* (2 isolates), *M. avium* subsp. *marseillense* (16 isolates), *M. intracellulare* (82 isolates), *M. intracellulare* subsp. *yongonense* (4 isolates), and *M. chimaera* (1 isolate). The MABC included three subspecies: *M. abscessus* (67 isolates), *M. bolletii* (2 isolates), and *M. massiliense* (45 isolates).

Next, we analyzed the geographical distribution of the three most frequent NTM species, and found that 114 MABC isolates were obtained from 6 provinces, with 113 strains distributed in southern China; in particular, 93 isolates were from Guangdong province. Further 108 MAC isolates were from 16 provinces (all 317 isolates were obtained from 21 provinces) with no noticeable regional differences. Although only 31 M*. kansasii* were obtained from 11 provinces, the regional distribution was wide.

### Drug susceptibility testing results

The distributions of the corresponding degrees of susceptibility, including MIC50/MIC90 values of RGM, are presented in Table 2. Amikacin was the most active drug against RGM. The resistance rate to amikacin was 4.69% (6/128), while the resistance rate to cefoxitin in RGM was 18.90% (24/127). For clarithromycin, 14.96% (19/127) of RGM were resistant at both day 3 and day 14, corresponding to acquired resistance to clarithromycin, while a total of 38.58% (49/127) of RGM were susceptible on day 3, but resistant on day 14, indicating inducible resistance to clarithromycin. The resistance rate to linezolid was 34.65% for RGM. Resistance rates to imipenem, tobramycin, doxycycline, cefepime, trimethoprim/sulfamethoxazole (TMP-SMX), minocycline, moxifloxacin, ciprofloxacin, ceftriaxone, and amoxicillin/clavulanic acid were high for RGM. As shown in Table 3, a comparison of drug susceptibility patterns among the major MABC species, *M. abscessus* and *M. massiliense*, indicated a significant difference in clarithromycin-inducible resistance rates (65.67% vs 2.22%) between the two species.

**Table 2:** Susceptibility of RGM to antimicrobial agents determined by the broth dilution method

Grouping/species	RGM (*n* = 128)										
Antimicrobial agent	Broth dilution ranges (μg/ml)	MIC50	MIC90	Susceptible		Intermediate		Resistant	S (%)	I (%)	R (%)
Antimicrobial agents and susceptibility breakpoints (MICs) for testing rapidly growing mycobacteriaa											
Amikacin	1‒64	8	16		≤ 16		32	≥ 64	93.75	1.56	4.69
Clarithromycin (3D)	0.06‒16	0.12	8		≤ 2		4	≥ 8	85.04	0	14.96
Clarithromycin (14D)		8	> 16						44.09	2.36	53.5
Imipenem	2‒64	> 64	> 64		≤ 4		8‒16	≥ 32	0	3.15	96.85
Linezolid	1‒32	16	> 32		≤ 8		16	≥ 32	33.07	32.28	34.6
Cefoxitin	4‒128	64	128		≤ 16		32‒64	≥ 128	6.30	74.8	18.90
Tobramycin	1‒16	8	> 16		≤ 2		4	≥ 8	13.39	12.60	74.02
Doxycycline	0.12‒16	> 16	> 16		≤ 1		2‒4	≥ 8	4.72	0	95.28
Moxifloxacin	0.25‒8	8	> 8		≤ 1		2	≥ 4	7.81	1.56	90.63
Ciprofloxacin	0.12‒4	> 4	> 4		≤ 1		2	≥ 4	11.02	0.79	88.19
Tigecycline	0.015‒4	1	2		-		-	-	-	-	-
TMP‒SMX	0.25/4.75‒8/152	> 8/152	> 8/152		≤ 2/38		-	≥ 4/76	4.72	-	95.28

*NTM* nontuberculous mycobacteria, *RGM* rapid growing mycobacteria, *SGM* slow growing mycobacteria, *TMP‒SMX* trimethoprim-sulfamethoxazole

^a^CLSI M24, 3rd ed. 2018 (2)

**Table 3:** Susceptibility of *Mycobacterium abscessus* and *M. massiliense* to antimicrobial agents determined by the broth dilution method

Grouping/species	*Mycobacterium abscessus* complex (*N* = 114)			*M. abscessus* (*n* = 67)			*M. massiliense* (*n* = 45)		
Antimicrobial agent	S (%)	I (%)	R (%)	S (%)	I (%)	R (%)	S (%)	I (%)	R (%)
Amikacin	94.74	1.75	3.51	92.54	1.49	5.97	100.00	0.00	0.00
Clarithromycin (3D)	85.09	0.00	14.91	82.09	0.00	17.91	91.11	0.00	8.89
Clarithromycin (14D)	42.98	2.63	54.39	13.43	2.99	83.58^a^	86.67	2.22	11.11^a^
Imipenem	0.00	1.75	98.25	0.00	0.00	100.00	0.00	2.22	97.78
Linezolid	32.46	34.21	33.33	29.85	34.33	35.82	35.56	35.56	28.89
Cefoxitin	4.39	78.95	16.67	4.48	76.12	19.40	4.44	84.44	11.11
Tobramycin	11.40	12.28	76.32	8.96	10.45	80.60	13.33	15.56	71.11
Doxycycline	1.75	0.00	98.25	1.49	0.00	98.51	2.22	0.00	97.78
Moxifloxacin	2.63	1.75	95.61	1.49	1.49	97.01	2.22	0.00	97.78
Ciprofloxacin	5.26	0.88	93.86	5.97	0.00	94.03	4.44	0.00	95.56
Tigecycline	-	-	-	-	-	-	-	-	-
TMP‒SMX	2.63	-	97.37	2.99	-	97.01	2.22	0.00	97.78

*RGM* rapid growing mycobacteria, *TMP‒SMX* trimethoprim-sulfamethoxazole, *CLSI* Clinical and Laboratory Standards Institute

^a^Significant difference between *M. abscessus* and *M. massiliense* was observed in resistance rate to clarithromycin (*P* < 0.01)

We also obtained MIC data for 189 SGM isolates. Data on the susceptibility of the 108 MAC isolates to 4 antimicrobial agents are presented in Table 4. Clarithromycin was the most active drug against MAC, with a 4.63% (5/108) resistance rate, while the resistance rate to amikacin was 10.19% (11/108). Clarithromycin and amikacin are the first-line antimicrobials to treat MAC infections, which showed excellent bacteriostatic effects. MAC isolates' resistance rates to the second-line antimicrobials, linezolid, and moxifloxacin, were 66.36% and 47.66%, respectively. Data on the susceptibility of 31 *M. kansasii* isolates to 9 antimicrobial agents is presented in Table 5. No *M. kansasii* isolates were resistant to clarithromycin, amikacin, or moxifloxacin. The rates of resistance to other first- and second-line antimycobacterial drugs was lower than 50%, other than those of doxycycline (77.42%, 24/31) and TMP-SMX (51.61%, 16/31). The susceptibility data of 50 SGM other than MAC and *M. kansasii* to 9 antimicrobial agents is presented in Table 6. The resistant rate of amikacin, clarithromycin and rifabutin is lower than 10%, and it is followed by moxifloxacin (26%, 13/50) and linezolid (38%, 19/50). As shown in Table 7, comparing drug susceptibility patterns among SGM, *M. kansasii* indicated the lowest resistant rate in linezolid and moxifloxacin, followed by SGM other than MAC and M. kansasii; MAC showed highest resistant rate in linezolid and moxifloxacin among SGM.

**Table 4:** Susceptibility of MAC to antimicrobial agents determined by the broth dilution method

Grouping/species	*Mycobacterium avium‒intracellulare* complex (*n* = 108)								
Antimicrobial agent	Broth dilution range (μg/ml)	MIC50	MIC90	Susceptible	Intermediate	Resistant	S (%)	I (%)	R (%)
Antimicrobial agents and susceptibility breakpoints (MICs) for testing MAC^a^									
First line									
Clarithromycin	0.06‒64	2	4	≤ 8	16	≥ 32	95.37	0	4.63
Amikacin	1‒64	16	64	≤ 16	32	≥ 64	71.30	18.52	10.19
Second line									
Linezolid	1‒64	32	64	≤ 8	16	≥ 32	14.81	18.52	66.67
Moxifloxacin	0.12‒8	2	8	≤ 1	2	≥ 4	15.74	37.04	47.22

*MAC* mycobacterium avium‒intracellulare complex, *CLSI* Clinical and Laboratory Standards Institute

^a^CLSI M24, 3rd ed., 2018 (2)

**Table 5:** Susceptibility of *Mycobacterium kansasii* to antimicrobial agents determined by the broth dilution method

Grouping/species	*M*. *Kansasii* (*n* = 31)								
Antimicrobial agent	Broth dilution ranges (μg/ml)	MIC50	MIC90	Susceptible	Intermediate	Resistant	S (%)	I (%)	R (%)
Antimicrobial agents and susceptibility breakpoints (MICs) for testing M. *kansasii*^a^									
First line									
Clarithromycin	0.06‒64	0.25	0.5	≤ 8	16	≥ 32	100	0	0
Rifampicin	0.12‒8	0.5	0.5	≤ 1	-	≥ 2	93.55	-	6.45
Second line									
Amikacin	1‒64	2	8	≤ 16	32	≥ 64	96.77	3.23	0
Ciprofloxacin	0.12‒16	2	4	≤ 1	2	≥ 4	32.26	41.93	25.81
Doxycycline	0.12‒16	8	16	≤ 1	2‒4	≥ 8	9.68	12.90	77.42
Linezolid	1‒64	2	4	≤ 8	16	≥ 32	96.77	0	3.23
Moxifloxacin	0.12‒8	≤ 0.12	0.25	≤ 1	2	≥ 4	96.77	3.23	0
Rifabutin	0.25‒8	≤ 0.25	0.5	≤ 2	-	≥ 4	96.77	0	3.23
TMP‒SMX	0.12/2.38‒8/152	4/76	> 8/152	≤ 2/38	-	≥ 4/76	48.39	-	51.61

*TMP‒SMX* trimethoprim-sulfamethoxazole, *CLSI* Clinical and Laboratory Standards Institute

^a^CLSI M24, 3rd ed. 2018 (2)

**Table 6:** Susceptibility of SGM other than MAC and *Mycobacterium kansasii* to antimicrobial agents determined by the broth dilution method

Grouping/species	SGM other than MAC and M. *kansasii* (*n* = 50)								
Antimicrobial agent	Broth dilution ranges (μg/ml)	MIC50	MIC90	Susceptible	Intermediate	Resistant	S (%)	I (%)	R (%)
Antimicrobial agents and susceptibility breakpoints (MICs) for testing SGM other than MAC and M. *kansasii*^a^									
Amikacin	1‒64	4	32	≤ 16	32	≥ 64	86	6	8
Ciprofloxacin	0.12‒16	4	> 16	≤ 1	2	≥ 4	12	24	64
Clarithromycin	0.06‒64	0.5	4	≤ 8	16	≥ 32	94	0	6
Doxycycline	0.12‒16	> 16	> 16	≤ 1	2‒4	≥ 8	8	10	82
Linezolid	1‒64	8	64	≤ 8	16	≥ 32	58	4	38
Moxifloxacin	0.12‒8	2	> 8	≤ 1	2	≥ 4	50	24	26
Rifampicin	0.12‒8	2	> 8	≤ 2	-	≥ 4	56	-	44
Rifabutin	0.25‒8	0.5	1	≤ 1	-	≥ 2	96	-	4
TMP‒SMX	0.12/2.38‒8/152	2	> 8	≤ 2/38	-	≥ 4/76	46	-	54

*SGM* slow growing mycobacteria, *MAC* *Mycobacterium avium*‒intracellulare complex, *CLSI* Clinical and Laboratory Standards Institute, *TMP‒SMX* trimethoprim-sulfamethoxazole

^a^CLSI M24, 3rd ed., 2018 (2)

**Table 7:** Percentage of drug resistance among different species of SGM

Grouping/species	MAC		*Mycobacterium kansasii*		Other SGM	
Antimicrobial agent	R (%, *n*/*N*)	MAC vs *M. kansasii*	R (%, *n*/*N*)	*M. kansasii* vs Other SGM	R (%, *n*/*N*)	MAC vs Other SGM
		p Value		*P* Value		*P* Value
Amikacin	10.19 (11/108)	0.12	0 (0/31)	0.16	8 (4/50)	0.88
Clarithromycin	4.63 (5/108)	0.35	0 (0/31)	0.28	6 (3/50)	1.00
Linezolid	66.67 (72/108)	< 0.01^a^	3.23 (1/31)	< 0.01^a^	38 (19/50)	< 0.01a
Moxifloxacin	47.22 (51/108)	< 0.01^a^	0 (0/31)	< 0.01^a^	26 (13/50)	0.01

*SGM* slow growing mycobacteria, *MAC* *Mycobacterium avium*‒intracellulare complex

^a^Significant difference was observed in resistance rate (*P* < 0.01)

## Discussion

This study demonstrates that, among all mycobacterial culture-positive pulmonary disease cases, 6.4% were NTM, based on nationwide surveillance of drug-resistant tuberculosis. Pulmonary NTM infection was more frequent in southern China, particularly southern coastal areas with high humidity. The most prevalent SGM was the MAC, which comprised seven subspecies, among which *M. intracellulare* was predominant and distributed widely across northern and southern China. The most prevalent RGM was the MABC, comprising three subspecies, with *M. abscessus* the predominant subspecies and mainly distributed in south China. The results of DST indicated that the drug-resistance spectrum varied greatly across different strains and subspecies. NTM showed relatively low resistance rates to macrolides and amikacin in vitro.

Distinguishing NTM from MTBC infection is of great clinical significance, as it can direct accurate and rapid clinical treatment [14–16]. The screening method used for NTM species is based on p-Nitrobenzoic acid, a time-consuming and challenging method, most reports from China can inhibit *M. tuberculosis* complex growth [17]. The laboratory diagnosis methods used to identify mycobacterial species have evolved over the decades [18]. The development of several extraction methods enhances the number of bacterial proteins available for MALDI-TOF MS identification. With the increasing amount of mycobacteria data available in commercial databases, MALDI-TOF MS technology has been implemented for NTM identification in many laboratories [8, 19]. Several studies have demonstrated that this method can achieve more than 95% agreement with results from DNA sequencing of variable genomic regions (including the *16S* rRNA, *hsp65*, *rpoB*, and *ITS* genes) [5, 20]. In our research, we obtained a 98.4% NTM detection rate and achieved 93.4% agreement with *16S* rRNA, *hsp65*, *ITS*, and *rpoB* gene sequencing. Although we could not identify NTM strains that were not contained in the Bruker MBT strains database, MALDI-TOF MS was able to identify the most clinically relevant NTM in a rapid, reliable, and inexpensive manner.

The overall NTM pulmonary infection rate was approximately 6% in our study, similar to that reported in a systematic review and meta-analysis of NTM infections, which demonstrated that the prevalence of NTM infections among patients with suspected tuberculosis was 6.3% in the mainland of China [7]. Our study showed the geographic variability in both the prevalence of NTM infections and mycobacterial species composition. A previous investigation in southern-central China demonstrated that the NTM infection rate is 4.0%, with the two most prevalent species the *M. avium-intracellulare* and *Mycobacterium chelonae-abscessus* complexes [6], while a report from Shanghai found an overall rate of NTM isolation from mycobacterial culture-positive patients of 5.9%, with *M. kansasii* the most frequently identified species, with an increasing trend from 3.0% in 2008 to 8.5% in 2012 [21]. In our study, the most frequent Shanghai province species was also *M. kansasii* (7/11 isolates), with a further increase in the NTM prevalence rate to 11% in 2013. In another study in Guangdong and Shanghai provinces, *M. intracellulare* was the most commonly isolated NTM in Shanghai, while *M. abscessus* was the most frequently isolated species in Guangzhou [22]. Some reports from eastern and northern China regions have demonstrated NTM prevalence rates of around 2.0%–3.0%, with *M. intracellulare* the predominant species, followed by *M. abscessus* [5, 21, 23]. In our study, NTM infection was more prevalent in southern than northern China and more frequent in eastern than western China. The most epidemic NTM species were MAC, which was widely distributed, and MABC which is mainly distributed in southeastern China. In addition, we isolated 16 *M. marseillense* strains of the MAC from sputum samples. Pulmonary disease caused by *M. marseillense* warrants increased attention, as it is infrequently reported [24, 25].

In addition to *Mycobacteria* spp. we also identified some acid-fast-staining-positive non-mycobacteria. As shown in Fig. 1, we randomly selected 60 non-mycobacteria from 286 contaminated or other species for species identification, including 15 *Gordonia*, 2 *Nocardia*, 2 *Streptococcus*, and 1 *Tsukamurella* (data not shown). The 15 *Gordonia* (comprising 8 *G. sputi*, 4 *G. bronchialis*, and 3 *G. rubripertincta*) isolates were distributed across nine provinces. Interestingly, the presence of *Gordonia* is consistent with a previous report from China [22]. Two *Nocardia* species, which often cause chronic lung disease, were isolated, as previously reported in China [23]. Besides, a case of *Tsukamurella* has previously been reported in Jiangxi province, Southern-central China [6]. In addition to NTM and MTBC infection, *Gordonia* and *Nocardia* species should also be tested for when using acid-fast staining to diagnose pulmonary disease.

We evaluated RGM and SGM's susceptibility from China to antimicrobials by 239 measuring MIC values using the RAPIDMYCOI and SLOWMYCOI Sensititre™ panels, according to CLSI protocol M24-A2. No such simple commercial kits for MIC measurement are available in China, despite the increase in patients with NTM infections, and information on drug susceptibility of NTM isolates is lacking. We mainly analyzed the vulnerability of MABC isolates, which were the most common clinical RGM isolates. Inducible macrolide resistance leads to differences in treatment outcome between patients with *M. abscessus* and *M. massiliense* infections. Consistent with previous reports [26, 28], we found that *M. abscessus* had a higher inducible resistance (65.67% vs 2.22%, *P* < 0.01) and acquired resistance (17.91% vs 8.89%, *P* = 0.2841) rates for clarithromycin than *M. massiliense*. These results further emphasize *M. abscessus* and *M. massiliense* subspecies identification's importance to inform appropriate clinical treatment using different strategies. Amikacin was the most active antimicrobial agent showed a 94.74% overall susceptibility rate, similar to the overall susceptible rate observed in previous studies from China and Australia [29, 30]; however, higher resistance rates, from 28.2 to 76.0%, have been observed in Japan and South Korea [15, 26]. After amikacin, cefoxitin was the second most effective antimicrobial agent with a 16.67% resistance rate, unlike South Korea results [18], where the second most effective antimicrobial agent was linezolid, but consistent with findings from Japan [15]. The resistance rate to cefoxitin was higher in *M. abscessus* (19.40%) than *M. massiliense* (11.11%). With a resistance rate of 33.33%, Linezolid could be used as an alternative therapy choice against RGM isolates. Given the high resistance rates to the other drugs tested in our study, they may not be appropriate for treatment of MABC infections; however, studies of clinical therapeutic effects are required.

For SGM, we mainly analyzed the susceptibility of the MAC and *M. kansasii*, the two most frequent SGM species. Consistent with previous studies [31], macrolides and amikacin showed excellent in vitro activity against MAC isolates, with 90% susceptibility. Patients with MAC pulmonary diseases are frequently administered a combination of clarithromycin, ethambutol, and rifampicin. However, it is suggested that the treatment with clarithromycin and ethambutol is not inferior to treatment with clarithromycin, ethambutol, and rifampicin for MAC lung disease [32]. Our data supported the two treatment regimens as their resistance rates to ethambutol and rifampicin in vitro were 58.33% and 91.67%, respectively. Some researchers have reported differential drug susceptibility patterns of *M. chimaera* and other members of the MAC [33]. In our study, we only obtained one *M. chimaera* strain. We compared the drug susceptibility patterns of the two most frequent species of MAC and found no significant difference between *M. intracellulare* and *M. marseillense*. As *M. marseillense* infections are rare in humans [24, 25, 34], our drug susceptibility data adds to this species' knowledge.

*M. kansasii* was the second most frequent SGM species with a high susceptibility rate to most first- and second-line antibiotics. These drug susceptibility patterns were markedly different from those reported by a previous study, which included DST of a total of 78 M*. kansasii* strains from 13 provinces of China [35]. Except for ethambutol (83.87% vs 20.5%), the resistance rates in our study were lower than those reported by the previous study [35], as follows: clarithromycin (0 vs 20.5%), amikacin (0 vs 5.1%), rifampicin (6.45% vs 56.4%), rifabutin (3.23%% vs 34.6%), moxifloxacin (0 vs 16.7%), and linezolid (3.23%% vs 32.1%). Our results are similar to those of a study using 85 M*. kansasii* isolates from eight countries in Europe and Asia [36]. All 85 M*. kansasii* isolates were susceptible to rifampicin, amikacin, rifabutin, moxifloxacin, and linezolid in this study. Although all 31 M*. kansasii* isolates included in our study were from 13 provinces in China, more isolates should be tested to evaluate drug susceptibility patterns of *M. kansasii*, given the relatively small number of strains and regional disparities.

No NTM were identified from some provinces in the northwestern region, likely due to the small sample size. Also, there were a limited number of strains of each species. We have prepared to collect more samples from these regions to complete our analysis of NTM infection and drug resistance status in China, and plan to evaluate NTM infection using the isolates collected during nationwide surveillance of tuberculosis.

## Conclusions

In this study, we analyzed the drug susceptibility patterns of all 317 NTM strains. More NTM pulmonary disease was observed in southern and coastal China. SGM was widely distributed, and more RGM is present in south and coastal China. The antimicrobial resistance spectrum of different NTM species was significant different, accurate species identification would be facilitated to NTM pulmonary disease treatment. Understanding NTM pulmonary infection will facilitate the development of TB treatment and control targets.

## Data Availability

The datasets used and/or analyzed during the current study are available from the corresponding author on reasonable request.

## Abbreviations

NTM: Nontuberculous mycobacteria
NTRL: National tuberculosis reference laboratory
MICs: Minimal inhibitory concentrations
CI: Confidence interval
SGM: Slow growing mycobacteria
RGM: Rapid growing mycobacteria
NTM-PD: NTM pulmonary disease
MTB: Mycobacterium tuberculosis
DST: Drug susceptibility testing
L-J: Lowenstein-Jensen
MALDI-TOF MS: Matrix-assisted laser desorption ionization–time of flight mass spectrometry
CLSI: Clinical and Laboratory Standards Institute
MTBC: Mycobacterium tuberculosis complex
MABC: Mycobacterium abscessus complex
MAC: Mycobacterium avium-intracellulare complex
TMP‒SMX: Trimethoprim-sulfamethoxazole
